# Feasibility of Mobile Single-Lead Electrocardiogram Screening for Atrial Fibrillation: Multicenter Cross-Sectional Study

**DOI:** 10.2196/92799

**Published:** 2026-09-10

**Authors:** Robert Fenske, Denise Rossa, Christian Butter, Felix Muehlensiepen, Jonathan Nübel, Daniel Meretz, Oliver Ritter, Sebastian Spethmann, Rene Mantke, Mario Zerbaum, Nico Draheim, Nicole Lindenberg-Krause, Anja Haase-Fielitz

**Affiliations:** 1 Heart Center Brandenburg, Brandenburg Medical School (MHB) Theodor Fontane, Ladeburger Str. 17, Bernau, Brandenburg, 16321, Germany, 49 3338-694-649, 49 3338-694-644; 2 Center for Health Services Research (ZVF-BB), Faculty of Health Sciences Brandenburg, Brandenburg Medical School (MHB) Theodor Fontane, Rüdersdorf, Brandenburg, Germany; 3 Department of Cardiology, Nephrology and Pneumology, Faculty of Health Sciences Bradenburg, Brandenburg Medical School Theodor Fontane, University Hospital Brandenburg, Brandenburg an der Havel, Germany; 4 Department of Cardiology, Angiology and Intensive Care Medicine, Campus Mitte, German Heart Center at Charité – University Medical Center Berlin, corporate member of Freie Universität Berlin and Humboldt-Universität zu Berlin, Berlin, Germany; 5 DZHK (German Centre for cardiovascular research), Partner site, Berlin, Germany; 6 Department of Surgery, University Hospital Brandenburg, Brandenburg Medical School Theodor Fontane, Brandenburg, Germany; 7 General practitioner Dr. Zerbaum & colleagues MVZ, Petersilienstraße 12, Brandenburg, Germany; 8 Family practice Nico Draheim, Fehrbelliner Str. 18, KST 19630044, Wichmannstrasse 18, Neuruppin, Germany; 9 General medical practice Dr. Lindenberg, Berliner Allee 18a, Werneuchen, Germany; 10 Institute of Social Medicine and Health System Research, Otto von Guericke University Magdeburg, Madgeburg, Germany

**Keywords:** atrial fibrillation, screening, mobile health, single-lead ECG, AliveCor, telemedicine, electrocardiogram

## Abstract

**Background:**

Atrial fibrillation (AF) is a major contributor of thromboembolic stroke. However, early detection remains challenging and is severely hindered by structural limitations in health care infrastructure. While current clinical guidelines recognize a 30-second single-lead electrocardiogram (ECG) as a standard diagnostic tool, the actual deployment and real-world utility of mobile screening technologies across health care settings remain poorly understood.

**Objective:**

The primary objective of this study was to evaluate the real-world feasibility of a mobile single-lead ECG for the detection of AF in routine inpatient and outpatient settings. Feasibility was evaluated by the proportion of successfully completed recordings, patient acceptance, and the occurrence and characteristics of technical challenges during deployment.

**Methods:**

This multicenter, cross-sectional pragmatic implementation study enrolled patients across 3 university hospitals and 3 primary care practices. Screening was performed using an US Food and Drug Administration (FDA)–approved single-lead ECG device (AliveCor KardiaMobile). Patients with an automated device reading indicating “Possible AF” and no prior diagnosis of AF underwent a confirmatory 12-lead ECG. Patient awareness and acceptance were evaluated using an exploratory, descriptive questionnaire.

**Results:**

A total of 237 patients were enrolled (194 inpatients and 43 outpatients). The device algorithm provided an automatic interpretation in 95.3% (41/43) of the outpatients and 85.6% (166/194) of inpatients, resulting in an overall automated detection of AF in 21.5% (51/237) of the cohort. The algorithm identified newly suspected AF in 1.7% (4/237) of patients without a prior AF diagnosis. Of these, 1 case underwent same-day confirmatory 12-lead ECG and was confirmed as AF. Technical challenges occurred in 16.0% (38/237) of all recordings, with a numerical higher rate observed in the inpatient setting (34/194, 17.5% vs 4/43, 9.3% in outpatients; *P*=.25). Only 6.3% (15/237) of patients reported regular use of telemedicine applications.

**Conclusions:**

Mobile single-lead ECG screening is feasible in routine inpatient and outpatient settings and identified a substantial prevalence of AF, which increased with age. The high detection rate underscores the potential of wearable ECG devices to facilitate early detection of AF. However, the absence of systematic confirmatory ECG testing and limited patient engagement with telemedicine applications highlight challenges in real-world implementation.

## Introduction

Atrial fibrillation (AF) is the most common cardiac arrhythmia and is associated with an increased risk of stroke, cognitive decline, and reduced quality of life. The global prevalence of AF has increased substantially over the past 3 decades, with an estimated 60 million cases worldwide [1]. Individuals with AF are more likely to be hospitalized for both cardiovascular and noncardiovascular causes [2]. With the aging population in Germany and globally, the burden of AF is expected to grow further. In Germany, the number of hospitals has decreased by 15% and hospital beds by 30% since the early 1990s [3]. In rural and underserved regions, access to health care remains limited, with travel times to hospitals or outpatient clinics exceeding 45 minutes [4]. Furthermore, the geographic density of specialized outpatient cardiologists in Brandenburg is significantly below the German national average, placing a disproportionate burden on primary care practitioners to manage complex chronic conditions such as AF [5].

Current cardiology guidelines recommend either a standard 12-lead electrocardiogram (ECG) or a single-lead ECG recording at least 30 seconds for the diagnosis of AF. In addition, opportunistic screening is also advised for patients aged 65 years and over [6]. Digital technologies such as telemedicine are transforming health care delivery by improving cross-sectoral care [7]. The increasing use of smartphones and wearables also offers a new, practical, and cost-effective way to improve AF screening and increase detection rates. These tools are accessible at any time, can be operated without prior medical knowledge, and are well-suited for documenting sporadic, short-term arrhythmias [8]. A recent study conducted in Canadian primary care settings demonstrated that opportunistic AF screening results in a significant reduction in downstream adverse health outcomes and future health expenditures [9]. However, while most validation studies of mobile single-lead ECG algorithms have been conducted under tightly controlled idealized conditions, their performance in real-world, pragmatic clinical settings remains poorly understood. There is a critical need to evaluate how these tools function in routine, cross-sectoral practice, where patient populations are heterogeneous, workflows are complex, and resources are limited.

The primary aim of this study was to assess the feasibility and real-world implementation of opportunistic AF screening using a mobile single-lead ECG device in diverse inpatient and outpatient care settings.

## Methods

### Study Design

This multicenter, cross-sectional study was conducted across 3 inpatient departments at the Brandenburg Medical School (Bernau, Brandenburg and Neuruppin) and 3 primary care practices. Patients were screened for AF using a mobile single-lead ECG (AliveCor KardiaMobile). The study was designed and implemented as part of a student-led research project within the Science Internship Program at the Brandenburg Medical School.

### Study Population

Eligible patients were adults aged 65 years or older, recruited on September 29, 2021, World Heart Day. Patients who did not provide written informed consent or were under 65 years of age were excluded from the study. No additional exclusion criteria were applied. Inpatients were recruited from the departments of cardiology, surgery, internal medicine and oncology at 3 sites: Bernau, Brandenburg and Neuruppin. Outpatients were recruited from 3 primary care practices of general practitioners at the same geographic locations.

### Primary and Secondary Outcomes

The primary outcome was to evaluate the feasibility and real-world implementation of opportunistic AF screening using a mobile single-lead ECG device, assessed by the proportion of successfully completed automated recordings.

Feasibility of the mobile single-lead ECG screening was assessed using 3 key indicators: the proportion of successfully completed recordings, patient acceptance, and the occurrence and characteristics of technical challenges during deployment. A successful recording was defined as a 30-second ECG with a clear, interpretable trace and no signal artifacts. Patient acceptance was evaluated through self-reported feedback collected via a structured questionnaire (Multimedia Appendix 1), including willingness to repeat the screening and perceived ease of use. Technical challenges including signal artifacts, unclassifiable tracing, and device-related issues were documented and categorized by type (eg, conductivity problems, patient movement, technical errors).

Secondary outcomes comprised an exploratory assessment of concordance between algorithm classifications and chart-documented AF history. Because a contemporaneous reference-standard ECG was not systematically performed in all participants, no formal diagnostic accuracy analysis was undertaken.

### Study Intervention and Data Collection

Screening was conducted using a commercially available, US Food and Drug Administration (FDA)–approved handheld single-lead ECG device (AliveCor KardiaMobile, version 5.35.1). Patients placed the index fingers of both hands onto the integrated electrodes for a 30-second recording. The mobile single-lead ECG was used as the primary screening tool for all patients. Patients who received an automated alert of “Possible AF” and had no prior diagnosis of AF underwent a confirmatory 12-lead ECG. A standard 12-lead ECG was not performed systematically across the entire cohort. Subsequent clinical management, including physician consultations and the initiation of oral anticoagulation therapy, were managed directly by the attending clinical staff on the same day, in accordance with standard care pathways.

No repeat ECGs were systematically performed. When a recording was flagged as “Unclassified” or “Unreadable,” clinical staff performed basic, immediate troubleshooting, including wiping the device sensors, repositioning the patient’s fingers, and asking the patient to relax or minimize movement. If these steps did not resolve the issue within a short time, the recording was accepted as inconclusive and not repeated.

Health literacy and digital readiness were assessed using a self-designed 12-item exploratory questionnaire. The instrument was conceptually informed by established frameworks, including the European Health Literacy Survey and the Digital Health Literacy Instrument. The questionnaire combined closed-ended items (multiple-choice and multicategorical checkboxes) to capture baseline demographics, pre-existing cardiac history, and potential physical or psychological barriers to technology adoption. Open-ended, free-text responses were intentionally included to explore patient awareness of AF symptoms, enabling qualitative insights into symptom recognition and health-related perceptions.

### Statistical Analysis

Normality of continuous variables was assessed graphically using histograms. Normally distributed data were compared using the *t* test or, in cases of heterogeneous variances, the Welch test. Comparisons of nonnormally distributed data were conducted using the Mann-Whitney *U* test or the Wilcoxon test (for paired data). Categorical variables were compared using the chi-square test. Exploratory performance metrics of the automated algorithm including sensitivity and specificity were calculated by cross-referencing algorithm outputs with established, chart-documented diagnoses of AF. A *P* value of <.05 was considered statistically significant. All data were analyzed using SPSS version 29.0 (IBM Corp).

### Ethical Considerations

The study was performed in compliance with the World Medical Association Declaration of Helsinki on Ethical Principles for Medical Research Involving Human Subjects and was reviewed by the Institutional Review Board of the Brandenburg Medical School (E-01-2020122). Informed consent was obtained prior to study participation from all individuals. Patients received detailed information regarding the objectives and procedures of the study, data handling, and storage practices, as well as potential health risks associated with or following the examinations. By consenting, they approved the collection, processing, and analysis of their personal data. At any point, and without the need to provide justification, participants retained the right to withdraw from the study, which would entail the erasure of all personal data and its copies. No financial or material incentives were offered for participation.

## Results

### Overview

On World Heart Day 2021, all patients hospitalized at the departments of cardiology, surgery, internal medicine and oncology, as well as all outpatients with scheduled appointments at 3 primary care practices, were invited to participate in the study. A total of 43 outpatients and 194 inpatients were enrolled. Using the KardiaMobile ECG, AF was detected in 13.9% (6/43) of outpatients and in 23.2% (45/194) of inpatients, resulting in an overall AF detection rate of 21.5% (51/237). Among these, 4 (1.7%) patients without a prior history of AF received the automated device interpretation indication “Possible AF” and were therefore considered newly suspected AF cases. All 4 patients were female. One of these cases underwent same-day 12-lead ECG confirmation, and anticoagulation therapy was initiated accordingly.

### Patients

Demographic characteristics, comorbidities, and medication use among inpatients and outpatients are shown in Table 1. The overall median age was 73 (IQR 66‐82) years and 48% (114/237) of patients were female. Inpatients were older and more frequently presented with heart valve disease and heart failure compared to outpatients (Table 1). The prevalence of known AF (91 inpatients, 6 outpatients) increased with age in both groups: from 18.7% (17/91) (65‐69 years) to 45.0% (41/91) (80‐89 years) among inpatients.

**Table 1.** Demographic data and comorbidities in outpatients and inpatients.

Variable	Outpatients (n=43)	Inpatients (n=194)	*P* value
Demographics, mean (IQR)			
Age (years)	70.0 (65.0-76.0)	75.0 (68.0-82.0)	.26
Heart rate (beats/min)	78.0 (70.0-85.0)	80.0 (70.0-89.0)	.81
CHA_2_DS_2_-VASc^a^ score (points)	3.0 (2.0-4.0)	4.0 (3.0-5.0)	.10
Comorbidities, n (%)			
Arterial hypertension	32 (74.4)	144 (74.2)	.98
Coronary heart disease	15 (34.9)	76 (39.2)	.60
Diabetes mellitus	17 (39.5)	63 (32.5)	.38
Heart failure	6 (14.0)	72 (37.1)	.003
Pre-existing atrial fibrillation	6 (13.6)	87 (45.1)	<.001
Prior myocardial infarction	4 (9.3)	18 (9.3)	>.99
Heart valve disease	5 (11.6)	89 (45.9)	<.001
Chronic kidney disease	8 (18.6)	49 (25.3)	.36
Peripheral vascular disease	15 (34.9)	94 (48.5)	.11
Asthma bronchial	3 (7.0)	11 (5.7)	.74
Chronic obstructive pulmonary disease	7 (16.3)	21 (10.8)	.32
Medication, n (%)			
Beta-blockers	17 (37.8)	129 (66.5)	<.001
AT-1 receptor antagonists	14 (32.6)	63 (32.5)	.99
ACE^b^ inhibitors	12 (27.9)	60 (30.9)	.70
Aldosterone antagonists	3 (7.0)	39 (20.1)	.04
Calcium channel blockers	7 (16.3)	49 (25.3)	.21
Diuretics	10 (23.3)	104 (53.6)	<.001
Neprilysin inhibitors (ARNI)^c^	0 (0)	12 (6.2)	.09
Statins	19 (44.2)	97 (50)	.49
Acetylsalicylic acid	13 (30.2)	57 (29.4)	.91
DOACs^d^	5 (11.6)	66 (34.0)	.004
Thyroid medication	7 (16.3)	26 (13.4)	.62

a CHA_2_DS_2_-VASc: congestive heart failure, hypertension, age, diabetes mellitus, prior stroke or TIA or thromboembolism, vascular disease, age, sex category.

b ACE: angiotensin-converting enzyme.

c ARNI: angiotensin receptor/neprilysin inhibitor.

d DOAC: direct oral anticoagulant.

Patients with AF were more likely to have additional cardiovascular comorbidities, including prior myocardial infarction, coronary heart disease, or heart failure, compared to patients without AF (Table 2). Patients with AF also received direct oral anticoagulants more frequently (particularly apixaban and rivaroxaban), as well as beta-blockers, angiotensin-converting enzyme inhibitors, aldosterone antagonists, and diuretics, compared to patients without AF.

**Table 2.** Demographic data and comorbidities in patients with and without atrial fibrillation detection using KardiaMobile.

Variables	Patients with sinus rhythm (n=147)	Patients with AF detection (n=51)	*P* value
Demographics			
Age (years), median (IQR)	72.0 (66.0-78.0)	78.0 (75.0-83.0)	.004
Age >65 years, n (%)	120 (81.6)	47 (92.2)	.04
Age >75 years, n (%)	50 (34.0)	38 (74.5)	<.001
Gender (female), n (%)	85 (57.8)	28 (54.9)	.82
Heart rate (beats/min)	76.0 (69.0-85.0)	87.0 (73.0-95.0)	.03
CHA_2_DS_2_-VASc^a^ score (points)	4.0 (2.0-5.0)	5.0 (4.0-5.0)	.002
Comorbidities, n (%)			
Arterial hypertension	101 (68.7)	42 (82.4)	.04
Coronary heart disease	48 (32.7)	23 (45.1)	.09
Diabetes mellitus	46 (31.3)	16 (31.4)	.93
Heart failure	34 (23.4)	28 (54.9)	<.001
Prior myocardial infarction	14 (9.5)	3 (6.0)	.44
Heart valve disease	43 (29.3)	32 (62.7)	<.001
Chronic kidney disease	29 (19.7)	16 (31.3)	.07
Peripheral vascular disease	61 (41.5)	25 (49.0)	.06
Asthma bronchial	7 (4.8)	3 (6.0)	.73
Chronic obstructive pulmonary disease	15 (10.2)	6 (11.8)	.72
Medication, n (%)			
Beta-blockers	81 (55.1)	43 (84.3)	<.001
AT-1 receptor antagonists	50 (34.0)	13 (25.5)	.29
ACE^b^ inhibitor	46 (31.3)	17 (33.3)	.72
Aldosterone antagonists	16 (10.9)	20 (39.2)	<.001
Calcium channel blockers	42 (28.6)	4 (7.8)	.003
Diuretics	58 (39.5)	33 (64.7)	.001
Neprilysin inhibitor	3 (2.0)	6 (11.8)	.04
Statins	72 (49.0)	23 (45.1)	.72
Acetylsalicylic acid	53 (36.1)	6 (11.8)	.001
DOACs^c^	25 (17.0)	37 (72.5)	<.001
Thyroid medication	18 (12.2)	6 (11.8)	.96
Laboratory parameters, median (IQR)			
Potassium (mmol/l)	4.28 (3.98-4.52)	4.19 (3.81-4.43)	.66
Sodium (mmol/l)	139.0 (137.0-141.0)	138.5 (136.0-140.6)	.79
Creatinine (µmol/l)	91.2 (64.0-106.0)	98.9 (75.0-111.0)	.39
Thyroid-stimulating hormone (µlU/ml)	2.1 (0.9-2.4)	1.7 (1.1-1.9)	.48

a CHA_2_DS_2_-VASc: congestive heart failure, hypertension, age, diabetes mellitus, prior stroke or TIA or thromboembolism, vascular disease, age, sex category.

b ACE: angiotensin-converting enzyme.

c DOAC: direct oral anticoagulant.

### Feasibility of the Mobile Single-Lead ECG Screening

**Figure 1.**
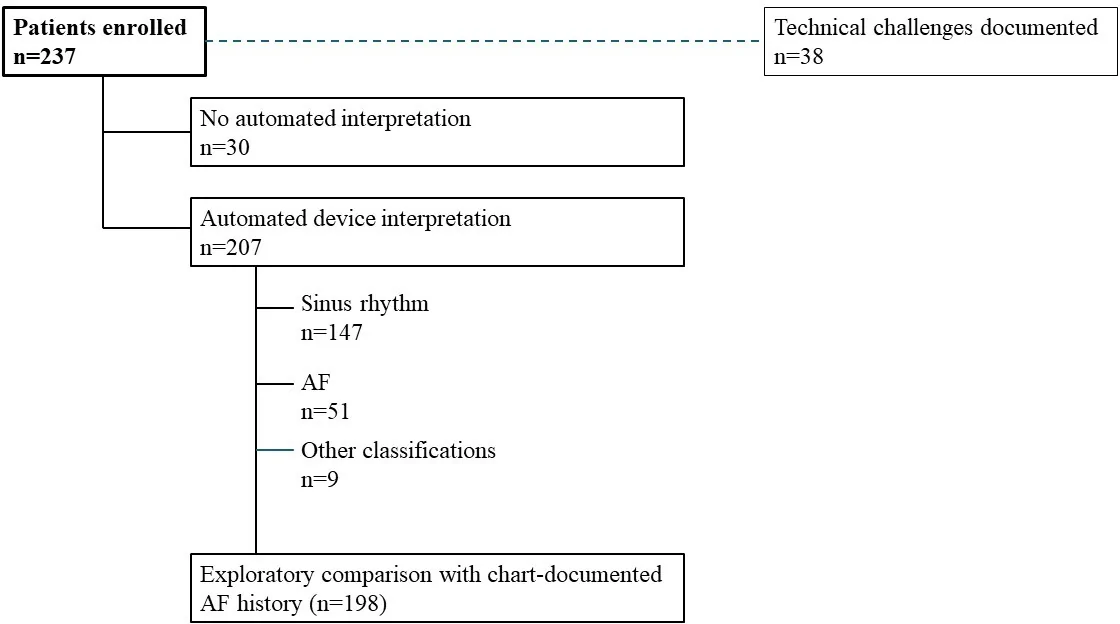
Flow of study participants and analysis populations. Overview of participant inclusion, automated device interpretation results, documented technical challenges, and the subset included in the exploratory comparison with chart-documented atrial fibrillation (AF) history.

The device algorithm provided an automated interpretation in 85.6% (166/194) of inpatient recordings and 95.3% (41/43) of outpatient recordings, demonstrating successful deployment in most patients (Figure 1). Technical challenges were documented in 38 (16.0%) recordings. These challenges did not necessarily prevent generation of automated device interpretation. The rate of technical challenges was slightly higher in the inpatient sector (34/194, 17.5%) compared to outpatients (4/43, 9.3%; *P*=.25). In 5 patients (2.1% of cases), no specific issue was documented. Technical challenges were primarily related to patient-related factors such as movement, poor electrode contact, or comorbidities and device-related issues including connectivity problems and software glitches. Among inpatients, the most frequent problems were related to comorbidities or compliance (11/194, 5.7%), technical errors (11/194, 5.7%), and conductivity issues (9/194, 4.6%). In outpatients, conductivity problems (4/43, 9.3%) were the most common reported issues.

### Exploratory Comparison With Chart-Documented AF

Of the 207 recordings that yielded an automated device interpretation, 198 were classified as either sinus rhythm (n=147) or AF (n=51) and were therefore included in the exploratory comparison with chart-documented AF history. The remaining recordings received alternative automated classifications and were excluded from this analysis.

### Use of Telemedicine and Patient Knowledge Regarding AF

**Figure 2.**
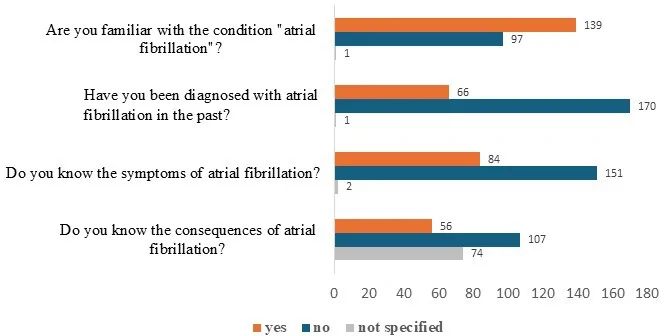
Patient knowledge about atrial fibrillation (absolute numbers).

In this study, 4.6% (9/194) of inpatients and 14.0% (6/43) of outpatients reported that they regularly use telemedicine applications such as health apps, telephone consultations, smartwatches, or contactless blood glucose monitoring. Patient acceptance of home-based mobile ECG screening was generally high; 56.5% (134/237) of participants expressed interest in using a 30-second ECG at home, and only 42.6% (101/237) declined. The most commonly reported reasons for refusal were visual impairment (29/237, 12.2%), skepticism toward digital health technologies (24/237, 10.1%), lack of interest (20/237, 8.4%), and concerns about technical difficulties (15/237, 6.3%). Patients’ knowledge about AF is shown in Figure 2.

Furthermore, among inpatients with AF documented in their medical records, 42.5% (37/87) were aware of their diagnosis. Of those, 89.2% (33/37) reported experiencing symptoms within the past 6 months.

Among outpatients, 6 of 43 (14.0%) reported a diagnosis of AF. The condition “atrial fibrillation” was recognized by 46.5% (20/43) of outpatients. Of these, 30.2% (13/43) were familiar with the symptoms, and 18.6% (8/43) were aware of potential complications, including stroke and myocardial infarction.

## Discussion

### Principal Findings

This multicenter study evaluated the feasibility and real-world performance of opportunistic AF screening using a mobile single-lead ECG (KardiaMobile) in routine inpatient and outpatient settings. Our findings demonstrate that mobile ECG screening is feasible in diverse clinical environments, with an overall AF detection rate of 21.5% (51/237), particularly among older adults. However, the study also reveals significant challenges in technical performance and patient-centered implementation, underscoring the gap between idealized validation and real-world application.

The observed prevalence of 21.5% substantially exceeds the estimated 1%‐2% prevalence of AF in the general German population and aligns with findings from high-risk cohorts. The elevated rate of AF is likely attributable to selection bias, as our cohort included hospitalized patients and patients visiting a general practitioner, groups inherently at higher risk for cardiovascular disease. The prevalence of AF increased with age among inpatients, reinforcing the value of age-targeted screening in older populations

In previous opportunistic screening studies using single-lead ECG, the incidence of newly diagnosed AF ranged from 0.49% [10] to 6.5% [11], depending on the baseline clinical risk profiles of the population. Our newly diagnosed rate of 1.7% is consistent with large-scale community screening initiatives, such as the study by Lowres et al [12], which reported a detection rate of 1.5% using a smartphone-based interface. However, in our study, only 1 case underwent confirmatory 12-lead ECG on the same day, reflecting both the pragmatic design of the study and the absence of systematic confirmatory testing for all screen-positive participants. Therefore, this figure should be interpreted as the rate of screen-detected suspected AF rather than confirmed newly diagnosed AF.

As expected, patients with AF had significantly higher median CHA_2_DS_2_-VASc (congestive heart failure, hypertension, age, diabetes mellitus, prior stroke or TIA or thromboembolism, vascular disease, age, sex category) scores than those in sinus rhythm. However, unlike the study by Lowres et al [12], we found no significant difference in risk scores between newly suspected and known AF cases. Notably, all our newly diagnosed patients were female, a finding that contradicts broader epidemiological data and is likely a statistical artifact due to the small sample size and the study’s recruitment focus on high-risk settings.

The growing prevalence of smartphone—already used by 65% of adults over 70 in Germany—provides a strong foundation for scalable digital screening [13]. The eBRAVE-AF study demonstrated that smartphone-based screening can more than double AF detection rates in individuals aged 50 years and older compared to routine care [14]. Similarly, the 12-month REHEARSE-AF study reported high patient adherence, with 90% of participants performing at least 1 ECG per week [15]. The STROKESTOP study, a multicenter, unmasked randomized controlled trial involving over 13,000 participants aged 75 and 76 years in Sweden, demonstrated that intermittent ECG screening could quadruple the detection rate of AF compared to single-time-point assessments [16]. STROKESTOP emphasized that high patient adherence and correct device use are critical for successful screening. In contrast to STROKESTOP, which required 14-day continuous monitoring, our study relied on a single 30-second recording. This difference underscores the trade-off between diagnostic sensitivity and operational feasibility. While longer monitoring increases detection rates, brief, opportunistic screening may offer a more scalable and sustainable model for implementation in diverse health care environments. While studies like STROKESTOP [16] and eBRAVE-AF [14] evaluate the efficacy of structured screening programs under controlled conditions, our study focused on the real-world feasibility and implementation of mobile ECG screening within routine clinical workflows, particularly in time-constrained, high-acuity environments.

Our study extends these findings by demonstrating that even brief, opportunistic screening with a mobile single-lead ECG can identify a substantial proportion of previously undiagnosed AF cases in real-world clinical practice without requiring prolonged monitoring or structured protocols. However, exploratory comparison of automated classifications with chart-documented AF history showed only moderate concordance for identifying patients with known AF. These findings differ from diagnostic validation studies conducted under controlled conditions but should not be interpreted as formal estimates of sensitivity or specificity because a universal reference-standard ECG was not applied [10,17,18]. Moreover, no repeat measurements were mandated for unclassified tracing, and results were interpreted in real time by clinical staff without independent expert review. Consequently, our data reflects the raw utility of the tool in an elderly and acutely ill cohort highly prone to muscle tremors and baseline ECG anomalies. These figures serve as exploratory metrics rather than definitive diagnostic validation, as a universal 12-lead ECG gold-standard reference was not implemented for all participants.

Technical challenges were common; 16.0% (38/237) of recordings were inconclusive, with a higher rate in inpatients than outpatients. These findings reflect the practical realities of deploying digital tools in acutely ill or frail patients, who may have tremors, reduced mobility, or cognitive impairments. Patient acceptance of home-based screening was high (56.5%), but 42.6% declined, citing visual impairment, skepticism toward digital health, lack of interest, and concerns about technical difficulties. These barriers are not trivial. They reflect real-world disparities in digital literacy and access, particularly among older adults. Notably, only 42.5% of inpatients with AF were aware of their diagnosis. While most patients reported experiencing symptoms like palpitations or dizziness, only 1.5% could link them to AF. This profound gap between symptom awareness and disease recognition highlights a critical opportunity for patient education and digital health interventions.

### Limitations

Several limitations must be considered. The study design may be susceptible to selection bias, as participants were recruited from high-risk inpatient and outpatient settings where AF prevalence is expected to be higher than in the general population. The authors were also not blinded to rhythm status. Real-time alerts from the KardiaMobile device were visible during screening, and confirmatory 12-lead ECGs were performed based on these results. This may introduce performance and detection bias. However, this study was designed as a pragmatic study, not a diagnostic validation study. The workflow reflects real-world practice, where clinicians are aware of patient context and use device alerts to guide care.

A further technical limitation was the inclusion of patients with permanent pacemakers or temporary pacing wires. In these patients, electronic pacing was frequently misinterpreted as signal artifacts, increasing the risk of false-positive AF detections or masking of underlying arrhythmias. Due to the absence of relevant data in the baseline dataset, these confounding factors could not be statistically adjusted for, potentially affecting the accuracy of the algorithm’s performance assessment. Only inpatients from surgery, cardiology, internal medicine, and oncology departments were included, which may limit the generalizability of the results. The readings from the mobile device were not systematically cross-checked by an independent panel of physicians. This workflow was deliberately chosen to realistically reflect daily clinical routine, including its common time and staffing constraints. The absence of a universal 12-lead ECG reference standard and independent expert review means that diagnostic performance metrics should be interpreted as exploratory, not definitive. We also have to acknowledge that our 12-item questionnaire used in the study is not a validated scale and lacks the depth of formal qualitative interviews. However, we intentionally chose this approach as an exploratory questionnaire to provide a practical, descriptive assessment of what patients know and how ready they are for digital health. Instead of offering deep qualitative insights, this survey serves a hypothesis-generating purpose. By showing real-world knowledge gaps and physical barriers, these descriptive findings give future studies a clear starting point to use detailed interview methodologies.

### Conclusions

This pragmatic feasibility study demonstrates that mobile single-lead ECG screening is feasible in routine inpatient and outpatient care and can be integrated into real-world clinical workflows. Technical challenges and barriers related to digital health literacy were common and should be considered in future implementation strategies. Because systematic confirmatory ECG testing was not performed in all participants, the study does not establish diagnostic accuracy, clinical benefit, stroke prevention, cost-effectiveness, or improved patient outcomes. Further prospective studies with rigorous validation and longitudinal follow-up are needed to evaluate the clinical impact and sustainability of mobile AF screening programs.

## Supplementary material

10.2196/92799 Multimedia Appendix 1 Questionnaire.

## Abbreviations

AF: atrial fibrillation
CHA_2_DS_2_-VASc: congestive heart failure, hypertension, age, diabetes mellitus, prior stroke or TIA or thromboembolism, vascular disease, age, sex category
ECG: electrocardiography
FDA: US Food and Drug Administration
