# Supplementary material for: Feasibility of Mobile Single-Lead Electrocardiogram Screening for Atrial Fibrillation: Multicenter Cross-Sectional Study
Source: JMIR Cardio. 2026 Sep 10;10:e92799. doi: 10.2196/92799 (PMC13561189; doi:10.2196/92799)
Supplement: Supplementary file 1 [file cardio-v10-e92799-s001.docx]

Questionnaire

| **Study Location** | *Bernau* | | | *Brandenburg an der Havel* | *Neuruppin* |
| --- | --- | --- | --- | --- | --- |
| Date: | O | | | O | O |
| **HealthCare Setting** | *Primary Care Practice* | | | *Inpatients Setting* | |
|  | O | | | O  O | |
| **Sex** | *Female* | *Male* | *Diverse* | | **Age** |
|  | O | O | O | |  |
| **Patient Questions** | | | *Yes* | | *No* |
| Are you familiar with the condition atrial fibrillation? | | | O | | O |
| Have you ever been diagnosed with atrial fibrillation? | | | O | | O |
| Are you familiar with the symptoms of atrial fibrillation? | | | O | | O |
| Are you familiar with the potential consequences of atrial fibrillation? | | | O | | O |
| If yes, which ones? | | | | | |
| **During the past six months, have you experienced any of the following symptoms: palpitations, rapid heartbeat, dizziness, chest pressure, or an irregular heartbeat?** | | | O Palpitations  O Rapid Heartbeat  O Dizziness  O Chest Pressure  O Irregular Heartbeat | | O |
| Do you use telemedical applications in your daily life? | | | O | | O |
| If yes, which ones? | | | O Telephone Consultation  O Video Consultation  O Health-Apps  O Remote Transmission of Medical Data | | |
| Other, not listed above (please specify) | | | | | |
| Would you like to use telemedicine services in your daily life? | | | O | | O |
| If yes, which ones? Please specify | | | | | |
| If no, what are the reasons? Please specify | | | | | |
